# Correlation Between Early Time-to-Event Outcomes and Overall Survival in Patients With Locally Advanced Head and Neck Squamous Cell Carcinoma Receiving Definitive Chemoradiation Therapy: Systematic Review and Meta-Analysis

**DOI:** 10.3389/fonc.2022.868490

**Published:** 2022-04-28

**Authors:** Christopher M. Black, Sam Keeping, Ali Mojebi, Karthik Ramakrishnan, Diana Chirovsky, Navneet Upadhyay, Dylan Maciel, Dieter Ayers

**Affiliations:** ^1^ Center for Observational and Real-World Evidence, Merck & Co., Inc., Kenilworth, NJ, United States; ^2^ Evidence Synthesis, PRECISIONheor, Vancouver, BC, Canada; ^3^ Center for Observational and Real-World Evidence, Former Employee of Merck & Co., Inc., Kenilworth, NJ, United States

**Keywords:** head and neck squamous cell carcinoma, surrogate endpoints, event-free survival, progression-free survival, overall survival, correlation analysis, chemoradiation therapy, systematic literature review

## Abstract

**Background:**

Overall survival (OS) is the most patient-relevant outcome in oncology; however, in early cancers, large sample sizes and extended follow-up durations are needed to detect statistically significant differences in OS between interventions. Use of early time-to-event outcomes as surrogates for OS can help facilitate faster approval of cancer therapies. In locally advanced head and neck squamous cell carcinoma (LA-HNSCC), event-free survival (EFS) was previously evaluated as a surrogate outcome (Michiels 2009) and demonstrated a strong correlation with OS. The current study aimed to further assess the correlation between EFS and OS in LA-HNSCC using an updated systematic literature review (SLR) focusing on patients receiving definitive chemoradiation therapy (CRT).

**Methods:**

An SLR was conducted on May 27, 2021 to identify randomized controlled trials assessing radiotherapy alone or CRT in the target population. Studies assessing CRT and reporting hazard ratios (HRs) or Kaplan-Meier data for OS and EFS were eligible for the analysis. CRT included any systemic treatments administered concurrently or sequentially with radiation therapy. Trial-level EFS/OS correlations were assessed using regression models, and the relationship strength was measured with Pearson correlation coefficient (R). Correlations were assessed across all CRT trials and in trial subsets assessing concurrent CRT, sequential CRT, RT+cisplatin, targeted therapies and intensity-modulated RT. Subgroup analysis was conducted among trials with similar EFS definitions (i.e. EFS including disease progression and/or death as events) and longer length of follow-up (i.e.≥ 5 years).

**Results:**

The SLR identified 149 trials of which 31 were included in the analysis. A strong correlation between EFS and OS was observed in the overall analysis of all CRT trials (R=0.85, 95% confidence interval: 0.72-0.93). Similar results were obtained in the sensitivity analyses of trials assessing concurrent CRT (R=0.88), sequential CRT (R=0.83), RT+cisplatin (R=0.82), targeted therapies (R=0.83) and intensity-modulated RT (R=0.86), as well as in trials with similar EFS definitions (R=0.87), with longer follow-up (R=0.81).

**Conclusion:**

EFS was strongly correlated with OS in this trial-level analysis. Future research using individual patient-level data can further investigate if EFS could be considered a suitable early clinical endpoint for evaluation of CRT regimens in LA-HNSCC patients receiving definitive CRT.

## Introduction

With nearly 750,000 new cases and 365,000 deaths each year, head and neck cancer is the eighth most common cancer worldwide (1). The locally advanced (LA) form of head and neck squamous cell carcinoma (HNSCC), defined as stages III-IVb based on the seventh edition of the American Joint Committee on Cancer (AJCC) staging system (2), is generally managed through various combinations of surgery, radiotherapy (RT), and systemic therapy (3). Specifically, chemoradiation therapy (CRT) that is concurrently administered, was shown to have a greater benefit than RT sequentially administered before or following chemotherapy in the meta-analysis of chemotherapy in head and neck cancer (MACH-NC) study (4–7). This, along with de-escalation RT strategies to lower toxicity rates, has led to improved survival in the subgroup of patients with human papillomavirus (HPV)-positive oropharyngeal squamous cell carcinoma (OPSCC); however, 5-year survival rates with CRT approaches still do not exceed 60% in the overall LA-HNSCC population, highlighting the unmet need to identify new treatment strategies that can increase the efficacy of CRT (8–10).

Overall survival (OS) is considered the most reliable outcome in oncology clinical trials and is one of the most valuable patient-centered outcomes that oncology treatments aim to improve (11, 12). The disadvantage of the OS outcome (with death as its only endpoint) in clinical trials is that it requires a large number of patients and an extended follow-up period to detect statistically significant differences, especially in earlier stages of cancer (13). In contrast, early time-to-event outcomes (e.g. event-free survival [EFS], progression-free survival [PFS], disease-free survival [DFS], and recurrence free survival [RFS]) additionally include disease progression in their definition, which occurs more frequently than death, especially in earlier cancer stages. As a result, these early time-to-event outcomes mature faster than OS, allowing for smaller sample sizes, shorter follow-up durations and earlier readouts for trials evaluating novel cancer therapies. However, for adoption of these alternate endpoints as primary trial endpoint, strong evidence and careful examination of the relationship between these early time-to-event outcomes and OS must be undertaken to establish a true surrogate relationship. If these endpoints can be shown to be surrogates for OS, more timely evaluations of the benefit-to-risk profile of experimental treatments and consequently earlier patient access to novel therapies can be achieved (14, 15).

A comprehensive examination of EFS and duration of locoregional control as potential surrogates for OS in patients with LA-HNSCC has previously been conducted in a 2009 study by Michiels et al. (16) In that study, individual patient-level data from 104 trials from four meta-analyses on hyperfractionated or accelerated RT and concomitant, induction, or adjuvant chemotherapy were analyzed at an individual level as well as at the trial level. The study concluded that OS was strongly correlated with EFS both at the individual level (range of correlation coefficient [R]: 0.82-0.90) and at the trial level (R=0.98 for RT, and range of 0.79-0.93 for CRT), and that at both levels, EFS was a stronger surrogate for OS compared to duration of locoregional control for patients receiving CRT. These results have broadly established the relevance and suitability of EFS as a surrogate for OS to assess the treatment effect of CRT in randomized controlled trials conducted in patients with LA-HNSCC who received locoregional treatments, including surgical interventions (16).

Targeted immunotherapies, such as inhibitors of programmed cell death protein 1, are being increasingly investigated in LA-HNSCC. Trials of these novel interventions, many of them still ongoing (17–21), are heterogeneous in terms of study design, population characteristics and primary endpoints. These trials were not captured in Michiels et al. (16), as the studies included in that analysis were published in or before 2000 (16). Furthermore, trial populations included in Michiels et al. (16) were a mix of surgery-eligible (e.g., receiving curative surgical intervention as part of their locoregional treatment) and surgery-ineligible patients. Given many novel treatments are evaluated in trials exclusively conducted in surgery-ineligible patients including time-to-event primary endpoints other than OS, it is of interest to further explore the association between EFS and OS in this population.

The current study aimed to assess the correlation between trial-reported OS and EFS using published clinical trial data among newly diagnosed patients with LA-HNSCC, with a special focus on trials evaluating definitive CRT without surgical intervention. Although patients with resectable tumors may still receive definitive CRT instead of surgery if they are suitable for organ preservation, many patients who receive definitive CRT are indeed ineligible for curative surgical interventions. For the purpose of completeness, CRT was defined as the combination of RT and any class of systemic therapies, including chemotherapy, targeted therapy or a combination of both. To minimize risk of bias, trials were identified through a comprehensive systematic literature review (SLR) using pre-defined criteria.

## Methods

### Study Selection

An SLR was conducted on May 27, 2021 to identify randomized controlled trials evaluating the efficacy of interventions for the treatment of newly diagnosed and untreated LA-HNSCC patients receiving definitive CRT (see **Supplementary Appendix A**). All types of interventions as recommended by clinical practice guidelines for this population were considered in the SLR (3, 22–27).. These included RT alone, or RT administered concurrently with systemic therapies (‘concurrent CRT’) or in the induction or adjuvant setting with systemic therapies (‘sequential CRT’). Any study that evaluated a primary intervention that included surgery was excluded.

Two reviewers independently reviewed abstracts and full-text publications to identify the trials meeting the predefined eligibility criteria (see **Supplementary Appendix A**). The process of study identification and selection was summarized with a Preferred Reporting Items for Systematic Reviews and Meta-Analyses (PRISMA) flow diagram (28). Data on study characteristics, interventions, patient characteristics, and outcomes for the final list of included studies were independently extracted by the two reviewers. At each stage, following reconciliation, a third reviewer was included to reach consensus on any remaining discrepancies. Data were stored and managed in a Microsoft Excel workbook.

Since CRT is the recommended regimen for patients in the target population (3, 22–27), trials of RT alone were not considered for the correlation analysis. As such, trials comparing CRT versus RT alone and trials comparing RT alone versus RT alone were excluded from the analysis. Furthermore, trials were required to report hazard ratios (HRs) or present Kaplan-Meier (KM) data for both OS and EFS. Trials reporting time-to-event outcomes similar to EFS (i.e. progression-free survival [PFS], disease-free survival [DFS], and recurrence-free survival [RFS]) were also included. This is consistent with the Michiels et al. study, where EFS broadly captured all time-to-event endpoints that included death due to any cause and disease progression in their definition (16).

### Correlation Analysis

The utility of an early clinical outcome as a good surrogate measure requires that the endpoints are likely to have a strong relationship to each other and that the change in surrogate outcome captures a large proportion of the treatment effect on meaningful outcomes such as OS (11, 29, 30). Among the methods developed to assess the predictive value of a surrogate outcome, trial-level surrogate validation in considered the most suited for regulatory approvals (11, 29). Such validation is performed by means of plotting a change in the surrogate (e.g. an early time-to-event outcome) against the change in the hard endpoint (e.g. OS) across several randomized controlled trials, with each trial serving as a single data point. A linear regression analysis is then performed to measure the correlation between change in the surrogate and change in the hard endpoint (11). Surrogacy conditions are considered to have been met if the intercept parameter (*β*_0_) is sufficiently close to zero and the slope parameter (*β*_1_) is significantly different from zero (additional details in **Supplementary Appendix B**).

Prior to the analysis, studies were examined for suitability for inclusion (as previously described). Study and patient characteristics were examined, and KM curves of both EFS and OS were examined to identify studies with potentially outlying characteristics. A weighted linear regression approach similar to prior studies (16, 31) was used to estimate the relationship between EFS and OS HRs. This approach modeled the relationship between the EFS ln(HR) and OS ln(HR) (additional details in **Supplementary Appendix B**). For each correlation analysis, the linear regression line was presented with its 95% confidence interval (CI), along with the estimated R value and its 95% CI, as well as the associated regression model equation. The same criteria used by Michiels et al. to determine a strong correlation (i.e. R > 0.75) were applied (16). Analyses were conducted using R version 4.0.4 (32).

### Model Scenarios

In the main analysis, data from all included CRT trials were incorporated into a simple regression model, regardless of their study or intervention characteristics, allowing for maximum statistical power. Additionally, several scenarios were explored to account for differences in study characteristics that may bias the correlation of EFS with OS. Follow-up duration was considered a potential source of bias by Michiels et al. (16), with over 91% of deaths occurring in the first 5 years of follow-up across the previously conducted meta-analyses (16). Timing of CRT was also found to interact with treatment effect in the MACH-NC study (7), with the benefit of concurrent CRT being significantly greater than sequential CRT. Although EFS was defined as the time from randomization to disease progression or death [consistent with the Michiels et al. study (16)], some trials included additional endpoints in their EFS definitions, which led to heterogeneity in EFS definitions. Non-cytotoxic targeted therapies (EGFR-inhibitors, immunotherapy) and intensity-modulated RT (IMRT), which both emerged after the Michiels et al. study, were also of interest. Lastly, subgroup of patients with p16-positive OPSCC was of interest given the tumor staging system and treatment algorithms for those patients have undergone major modifications since the publication of the Michiels et al. analysis, turning that subgroup into a separate entity in this disease area (3, 33).

Based on the above, the explored scenarios included models with interaction terms (details in **Supplementary Appendix B**) for trial maximum follow-up duration (>5 versus ≤5 years) and timing of the CRT (0 versus ≥1 sequential CRT treatment arm). The surrogacy relationship was further assessed within subsets of trials comparing alternative concurrent CRT regimens and trials comparing sequential CRT versus concurrent CRT. Furthermore, an analysis was conducted within a subset of trials where EFS was consistently defined as time from randomization/treatment initiation to death due to any cause or recurrence/disease progression, whichever came first.

With concurrent RT + cisplatin being recommended as treatment of choice for the target population based on high-quality evidence (3, 22–27), further analyses explored the EFS/OS correlation within all trials of concurrent RT + cisplatin, trials comparing concurrent CRT versus concurrent RT + cisplatin, and trials comparing sequential CRT versus concurrent RT + cisplatin. A subgroup analysis focused on trials evaluating targeted therapies in at least one treatment arm. Lastly, the subset of studies that reported relevant outcomes in patients with p16-positive OPSCC were analyzed although an initial review of the evidence base revealed there were few trials in this category; therefore, this was considered an exploratory scenario only.

### Model Validation

Each model was cross-validated using a leave-one-out analysis. A new regression model was fit with that trial removed, using only the data from the remaining trials. The outcome of the removed trial was then predicted from its observed EFS and trial size using this new regression model. This predicted OS was compared with its observed value and reported 95% CI. Predicted values that differed substantially from the observed values were indicative of a poor fit to the model. Additionally, a substantial difference indicated that the study in question may have had an undue influence on the overall model. The results from the leave-one-out analyses were used to assess the robustness of the overall models, to identify studies that differed from others, and to select the most appropriate model.

## Results

### Eligible Studies

**Figure 1:**
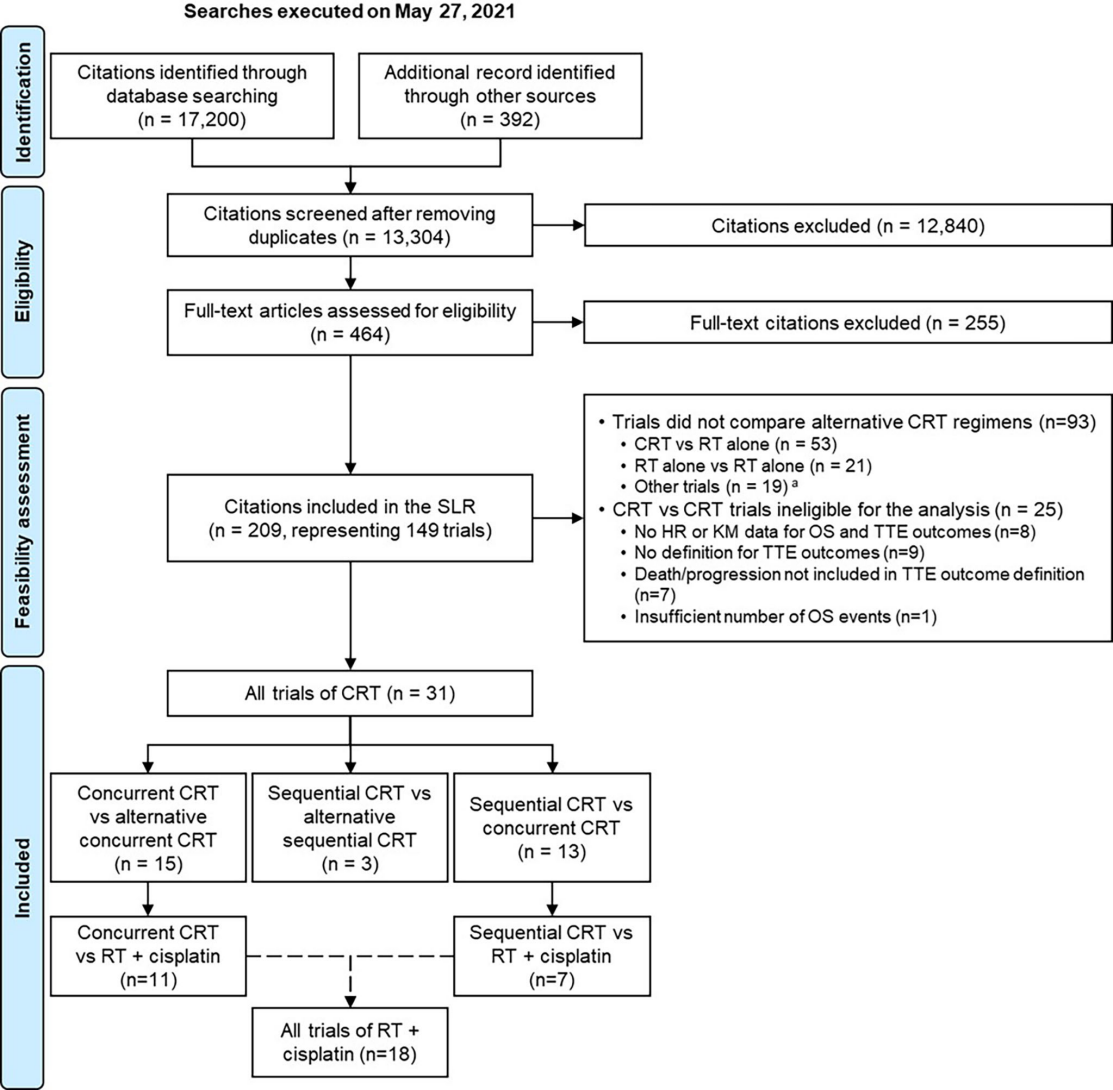
PRISMA flow diagram for the systematic literature review and the meta-analyses. CRT was defined as the combination of RT and any class of systemic therapies, including chemotherapy, targeted therapy or a combination of both. ^a^Trials comparing different doses/schedules of the same CRT regimen; to one another. HR, hazard ratio; KM, Kaplan-Meier; OS, overall survival; PRISMA, Preferred Reporting Items for Systematic Reviews; RT, radiotherapy; SLR, systematic literature review; TTE, time-to-event.

The SLR included 209 citations, corresponding to 149 unique randomized controlled trials in the target population (**Figure 1**). Of these, 93 trials did not compare alternative CRT regimens to one another and were therefore excluded from the analysis. Of the remaining 56 trials of CRT, 31 studies (34–64) met the eligibility criteria and were included in the analysis (see **Supplementary Appendix C**). The remaining 25 studies were excluded because they did not meet the pre-specified study eligibility criteria of the correlation analysis (24 trials) or had very few OS events in their presented KM data [*post-hoc* decision made to exclude one trial (65) for this reason] (see **Supplementary Appendix D**).

Of the 31 included trials, 13 were in phase II, 16 in phase III, and one was a phase II/III study (one did not report trial phase). Five trials were double-blinded and 23 were open-label (three did not report on masking). All trials were conducted in multiple centers, except for three single-center studies. Maximum follow-up duration was ≤5 years and >5 years in 19 and 11 trials, respectively. RT dosage generally ranged between 60 Gy and 70 Gy, with around half of the trials using current RT modalities such as 3D conformal RT and intensity-modulated RT (IMRT). Eighteen trials compared concurrent RT + cisplatin to either a concurrent CRT regimen (11 trials) or a sequential CRT regimen (seven trials). Besides cisplatin, systemic therapies that were often used in combination with RT included cytotoxic agents (e.g. hydroxyurea, docetaxel, 5-FU) and targeted therapies (e.g. cetuximab, gefitinib). Of note, 16 trials evaluated targeted therapies: two investigated immunotherapies (pembrolizumab and avelumab) and 14 investigated EGFR inhibitors (mostly cetuximab).

Early time-to-event outcomes of interest (collectively referred to as EFS in the current analysis) were labelled ‘PFS’ in 25 trials, ‘DFS’ and ‘failure-free survival’ in two trials each, and ‘RFS’ and ‘EFS’ in one trial each. Among the included trials, 22 had consistent outcome definitions that only included disease progression and death due to any cause as endpoints; in the remaining nine trials, other endpoints (e.g. second primary malignancy, residual disease left behind after neck dissection) were additionally included in the definitions. Furthermore, fourteen trials used RECIST (v1.0, v1.1, or the modified version) (66) to measure response/disease progression, and five used World Health Organization (WHO) criteria (original criteria or modified versions) (67).

### Correlation Analyses

The association between EFS and OS was moderate to strong in all correlation analyses, with the surrogacy conditions (a statistically significant slope and a non-significant intercept) being met in all scenarios except for the analysis of sequential CRT versus concurrent CRT trials.

**Figure 2:**
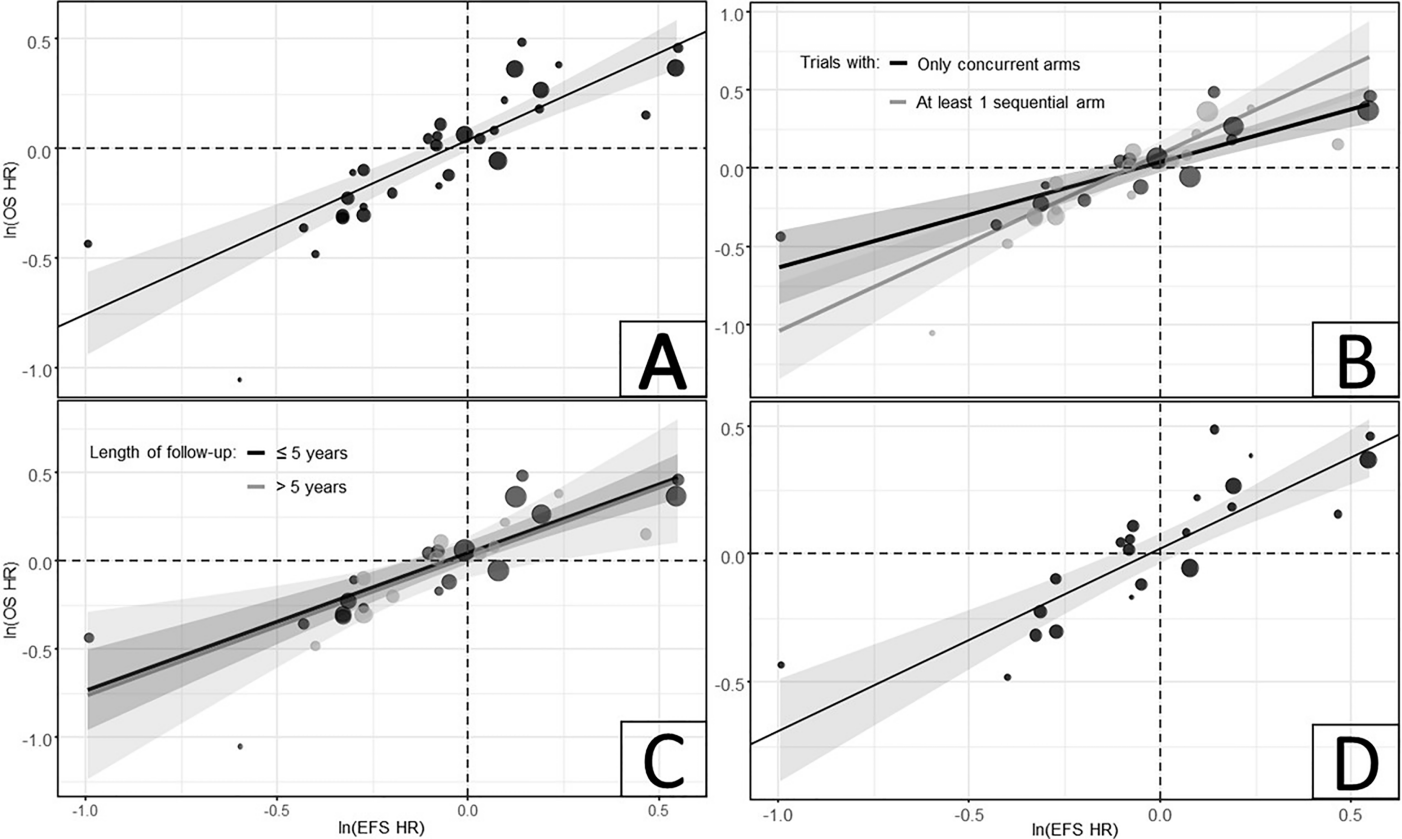
Relationship between ln(HRs) of EFS and OS in **(A)** all CRT trials; **(B)** Model with interaction term for timing of CRT; **(C)** Model with interaction term for length of follow-up duration; and **(D)** subset of trials with matching EFS definitions, only including death and disease progression as endpoints. CRT was defined as the combination of RT and any class of systemic therapies, including chemotherapy, targeted therapy or a combination of both. CI, confidence interval; CRT, chemoradiation therapy; EFS, event-free survival; HR, hazard ratio; OS, overall survival.

In the analysis of all CRT trials (31 trials), the estimated R was 0.85 (95% CI, 0.72-0.93), showing a strong correlation between ln(HR) of EFS and OS across all CRT trials (**Table 1**; **Figure 2A**). The observation in the bottom left corner of the regression model figure belongs to the Lim et al. (49) trial, which, due to its relatively small size, did not have a meaningful influence on the model.

**Table 1:** Estimated correlations between EFS and OS.

Scenario	N	R (95% CI)	Slope (95% CI)	Intercept (95% CI)
All CRT trials	31	0.85 (0.72 - 0.93)	0.79 (0.61 - 0.98)	0.04 (-0.01 - 0.09)
Interaction term for CRT timing				
Trials with concurrent CRT arms only	15	0.88 (0.66 - 0.96)	0.68 (0.46 - 0.89)	0.04 (-0.03 - 0.10)
Trials with at least one sequential CRT arm	16	0.88 (0.68 - 0.96)	1.14 (0.53 - 1.75)	0.09 (-0.08 - 0.26)
Interaction term for follow-up time^a^				
Maximum follow-up duration ≤5 years	19	0.86 (0.66 - 0.94)	0.79 (0.57 - 1.00)	0.05 (-0.02 - 0.11)
Maximum follow-up duration >5 years	11	0.81 (0.41 - 0.95)	0.79 (0.02 - 1.56)	0.02 (-0.17 - 0.22)
Trials with matching outcome definitions^b^	22	0.87 (0.71 - 0.95)	0.71 (0.52 - 0.90)	0.02 (-0.04 - 0.08)
Trials of concurrent CRT regimens				
Trials comparing alternative concurrent CRT regimens	15	0.88 (0.66 - 0.96)	0.67 (0.45 - 0.90)	0.04 (-0.03 - 0.10)
Sequential CRT versus concurrent CRT trials	13	0.83 (0.50 - 0.95)	1.01 (0.55 - 1.47)	0.11 (0.01 - 0.20)
Trials of RT + cisplatin				
All trials of concurrent RT + cisplatin	18	0.82 (0.58 - 0.93)	0.66 (0.42 - 0.91)	0.05 (-0.02 - 0.12)
Concurrent CRT versus concurrent RT + cisplatin trials	11	0.83 (0.46 - 0.95)	0.61 (0.30 - 0.92)	0.06 (-0.04 - 0.15)
Sequential CRT versus concurrent RT + cisplatin trials	7	0.94 (0.66 - 0.99)	1.56 (0.93 - 2.19)	0.05 (-0.04 - 0.14)
Trials of targeted therapies	16	0.83 (0.56 - 0.94)	0.78 (0.48 - 1.09)	0.05 (-0.03 - 0.14)
Trials of IMRT	18	0.86 (0.66 - 0.95)	0.70 (0.48 - 0.92)	0.02 (-0.05 - 0.09)
Trials in p16-positive OPSCC subgroup^c^	5	0.2 (-0.83 - 0.92)	0.16 (-1.23 - 1.55)	0.30 (-0.41 - 1.01)

CRT was defined as the combination of RT and any class of systemic therapies, including chemotherapy, targeted therapy or a combination of both. Surrogacy conditions were met in all scenarios except for the analysis of sequential CRT versus concurrent CRT trials. Surrogacy conditions were defined as an intercept point estimate that is not statistically significantly different from zero AND a slope point estimate that is statistically significantly different from zero.

a Maximum follow-up duration was not reported in Bourhis 2020 (GORTEC 2015-01); therefore, that trial was not included in this model.

b Time-to-event outcome definitions only included disease progression and death due to any cause as endpoints.

c Exploratory analysis.

CI, confidence interval; EFS, event-free survival; IMRT, intensity-modulated radiotherapy; OS, overall survival; OPSCC, oropharyngeal squamous cell carcinoma.

In the model with an interaction term for timing of the CRT, the estimated correlations for trials with concurrent CRT arms only (15 trials, R = 0.88 [95% CI: [0.66-0.96]) and those with at least one sequential CRT arm (16 trials, R = 0.88 [95% CI: 0.68-0.96]) indicated a strong correlation between EFS and OS in these trial subsets (**Table 1**; **Figure 2B**). Similarly, in the model with an interaction term for maximum length of follow-up, the estimated correlations for the ‘≤5 years’ (19 trials, R = 0.86 [95% CI: 0.66-0.94]) and ‘>5 years’ (11 trials, R = 0.81 [95% CI: 0.41-0.95]) subsets of studies showed strong correlations between EFS and OS (**Table 1**; **Figure 2C**). Among the trials with matching EFS definitions (22 trials), a slightly stronger correlation was observed (R = 0.87 [95% CI: 0.71-0.95]) compared to the analysis of all CRT trials (**Table 1**; **Figure 2D**).

Consistent with the above results, analyses of trials comparing alternative concurrent CRT regimens (15 trials, R = 0.88 [95% CI: 0.66-0.96]) and those comparing sequential CRT versus concurrent CRT (13 trials, R = 0.83 [95% CI: 0.50-0.95]) showed strong correlations between EFS and OS. This correlation remained strong among trials of RT + cisplatin (18 trials, R = 0.82 [95% CI: 0.58-0.93]), as well as within subsets of trials comparing concurrent CRT regimens (11 trials, R = 0.83 [95% CI: 0.46-0.95]) or sequential CRT regimens (seven trials, R = 0.94 [95% CI: 0.66-0.99]) versus RT + cisplatin (**Table 1**).

Finally, results from the analyses of studies evaluating targeted therapies (16 trials, R = 0.83 [95% CI: 0.56-0.94]) and those evaluating IMRT (18 trials, R = 0.86 [95% CI: 0.66-0.95]) were also similar to the other scenarios, with strong correlations observed within both subsets of trials (**Table 1**).

Only five trials met the eligibility criteria to be included in the exploratory analysis within the p16-positive OPSCC population: two trials were conducted entirely in the HPV-positive population and three reported outcomes for the target subgroup (sample sizes of 34, 18, and 32 patients). Results from this exploratory analysis (R = 0.2 [95% CI: -0.83-0.92]) were inconclusive because of the large 95% CI around the regression line due to both the small number of trials included in the analysis and the small sample sizes of the p16-positive subgroups (**Table 1**).

### Analysis Validation

Results from the leave-one-out analysis of the model including all CRT trials (see **Supplementary Appendix E**) showed that the predicted ln(OS HR) in the cross-validation analyses always fell within the observed 95% CIs (where reported). Furthermore, predictions were often close to the observed values reported for each trial, indicating that the regression models generally provided acceptable estimates when predicting OS in terms of ln(HR). This indicated that no single trial had undue influence on the predictive power of the regression model. Results from the leave-one-out validation models from the remaining correlation analyses were consistent with the analyses of all CRT trials (data not shown).

## Discussion

Curative therapeutic options are limited for surgery-ineligible patients with LA-HNSCC, highlighting an unmet need for novel interventions for the treatment of this population. Identifying early clinical outcomes that can be surrogates for OS is key to lowering the cost and duration of trials and, thereby, facilitating patient access to those novel treatments and improving clinical outcomes at a faster pace. The advantage of early time-to-event outcomes such as EFS or PFS in randomized controlled trials is that they take less time to mature relative to OS, thereby providing an earlier indication of likely treatment benefit(68–70). Additionally, they are not as affected by post-progression treatments, which can affect estimates of treatment effects on OS and confound the results (70, 71). For example, salvage surgery for persistent/recurrent disease (e.g. laryngectomy) can affect post-progression survival, possibly diluting the treatment effect on OS, with high complications rates and morbidity (10, 72, 73). These post-progression treatments are not currently included in the definition of time-to-event outcomes such as EFS or DFS in oncology trials (12), including the studies identified in our evidence base. Based on all the above, many phase III oncology studies have therefore adopted these alternate endpoints as the primary endpoint of the trial (11). However, it remains an ongoing debate as to whether there is strong evidence to suggest that these early time-to-event outcomes can be treated as surrogates for OS. Careful examination of the relationship between the early and final endpoints must be undertaken to establish a true surrogate relationship.

Results from the Michiels et al. study have broadly established the relevance and suitability of EFS as a surrogate for OS to assess the treatment effect of RT and chemotherapy in randomized controlled trials conducted in patients with LA-HNSCC (16). Our analysis followed the approach taken by that study, while complementing it by including additional trials and focusing on the subgroup patients receiving definitive CRT. The current study incorporated a comprehensive SLR conducted as early as May 2021. As such, compared to Michiels et al. (16), our evidence base additionally included trials published after 2000, which evaluated novel treatments such as immunotherapies and other targeted therapies used as part of definitive CRT in LA-HNSCC patients who do not receive surgery. In anticipation that the included clinical trials generally did not start after 2017, we used the disease staging criteria presented in the 7^th^ edition of the AJCC manual (as opposed to the 8^th^ edition, which was implemented in January 2018) to define the LA disease. Therefore, none of the studies were, for example, excluded based on the new staging system presented in the 8^th^ edition for p16-positive OPSCC. Once the SLR was completed, all of the included studies were found to have a start date prior to 2018, as initially expected.

EFS in our study additionally included other endpoints such as PFS, DFS, and RFS, because these early time-to-event outcomes have been the primary endpoints of many trials conducted in patients who receive definitive CRT, including studies of immunotherapy and other novel treatments (17–21). While these varied based on event definition (e.g. death, disease progression, second primary cancer, and surgery), all of them included death and disease progression as common events, per the study eligibility criteria of the analysis. Due to a higher percentage of disease progression events in LA-HNSCC compared to surgical events or second primary cancer events, inclusion of other time-to-event outcomes in the EFS definition may be less impactful in determining correlation between EFS and OS.

Results of the current study, while limited to trial-level analyses and being conducted in a slightly different population, support the Michiels et al. (16) results. In the main analysis inclusive of all CRT trials, the correlation coefficient (95% CI) was 0.85 (0.72-0.93), suggesting a strong correlation between EFS and OS. Due to differences in study characteristics, the surrogacy relationship was further evaluated in subsets of trials with similar length of follow up, CRT timing, outcome definition, intervention class, and RT modality. The correlation coefficient in these scenarios always remained above 0.82, suggesting that a similar relationship existed in all subsets. Specifically, CRT timing did not influence the strength of the correlation between EFS and OS. As the number of studies decreased within the analyzed subsets of trials, the 95% CIs became much wider, indicating greater uncertainty. In addition to the consistently strong correlations (estimated R ranging between 0.81 and 0.94), the expected trial-level surrogacy relationships (an intercept sufficiently close to zero and a slope significantly different from zero) were met in all scenarios, with the exception of the model for trials that compared sequential CRT versus concurrent CRT. Lastly, a leave-one-out cross-validation strategy was taken, similar to Michiels et al. (16), in order to ensure the robustness and accuracy of the predictive models. In the leave-one-out analyses, predictions generally fell within the reported CIs.

While the OS-EFS correlation was analyzed in a number of trial subsets based on major study, intervention and outcome characteristics, further scenarios could be explored in the future. For example, it may be of interest to evaluate the OS-EFS correlation in patients with p16-positive OPSCC given new staging and treatment algorithms have been developed for this particular population over the last two decades (3, 33). Among the trials of CRT included in the current analysis, only five reported outcomes of interest in patients with p16-positive OPSCC, precluding conclusive results for the exploratory analysis in that subpopulation. Future research can potentially focus on evidence bases other than the one synthesized in the current study; for example, the OS-EFS correlation could be further explored in trials evaluating RT alone in p16-positive patients, particularly those using the de-escalation treatment strategy with lower toxicity rates (74). Such trials, however, were not directly related to the research questions of the current analysis and were not included in our SLR.

This analysis was subject to some limitations. Our study lacked individual patient-level data. A proper assessment of surrogacy requires that there is a relationship between the two outcomes at the patient-level as well as a relationship between treatment effects at the trial-level (75). Furthermore, patient-level data allows to account for differences in baseline patient characteristics between studies (e.g. age, gender, smoking status, HPV status, ECOG scores) that could influence the EFS and OS relationship. In the absence of individual patient-level data, we were only able to assess the trial-level surrogacy relationships using aggregate data. Furthermore, EFS and OS correlation may vary by the type of event. For instance, distant metastatic events may be more strongly correlated to OS compared to a locoregional event given disease prognosis in LA-HNSCC. This was shown in the Michiels et al. study, where EFS (inclusive of locoregional and distant metastatic events) was a stronger surrogate for OS compared to duration of locoregional control for patients receiving CRT. Due to lack of patient-level data we could not assess this aspect in our study. Finally, the majority of the included CRT studies were completed prior to approval of immunotherapies in recurrent and/or metastatic HNSCC (R/M HNSCC) (76–80). The improvement in OS from use of immunotherapies in R/M HNSCC, might influence the correlation between EFS and OS. Thus, it is important that future correlation research focusses on more recent LA-HNSCC trials (i.e. initiated or completed after approval of immunotherapies in R/M HNSCC) in which patients are eligible to receive immunotherapies after failure of CRT.

Despite some limitations, the trial-level analyses were conducted on a dataset that was obtained from a rigorous SLR, and the models employed were assessed for appropriateness. The cross-validation procedure showed that the correlation models were generally able to predict OS HRs with acceptable accuracy. In conclusion, EFS was strongly correlated with OS in this trial-level analysis. Future research using individual patient-level data can further investigate if EFS could be considered a suitable early clinical endpoint for evaluation of CRT regimens in LA-HNSCC patients receiving definitive CRT.

## Data Availability Statement

The original contributions presented in the study are included in the article/**Supplementary Material**. Further inquiries can be directed to the corresponding author.

## Author Contributions

Conception/design was performed by SK, DA, AM, CMB, DC, KR, and NU. Collection and/or assembly of data was performed by SK, DA, and AM. Data analysis and interpretation were performed by SK, DA, DM, AM, CMB, DC, KR, and NU. Manuscript writing was performed by AM and KR. Final approval of manuscript was provided by SK, DA, AM, DM, CMB, DC, KR, and NU. All authors contributed to the article and approved the submitted version.

## Funding

Funding for the study was provided by Merck Sharp & Dohme Corp., a subsidiary of Merck & Co., Inc., Kenilworth, NJ, USA.

## Conflict of Interest

KR is an employee of Merck Sharp & Dohme Corp., a subsidiary of Merck & Co., Inc., Kenilworth, NJ, USA. NU was employed by Merck Sharp & Dohme Corp., a subsidiary of Merck & Co., Inc., Kenilworth, NJ, USA, at the time the research was conducted. CB and DC are employees of Merck Sharp & Dohme Corp., a subsidiary of Merck & Co., Inc., Kenilworth, NJ, USA and stockholders of Merck & Co., Inc., Kenilworth, NJ, USA. SK, DA, AM, and DM are employees of PRECISIONheor, which received funding for this study.

## Publisher’s Note

All claims expressed in this article are solely those of the authors and do not necessarily represent those of their affiliated organizations, or those of the publisher, the editors and the reviewers. Any product that may be evaluated in this article, or claim that may be made by its manufacturer, is not guaranteed or endorsed by the publisher.

## Abbreviations

AJCC, American Joint Committee on Cancer; CI, confidence interval; CRT, chemoradiation therapy; DFS, disease-free survival; ECOG, Eastern Cooperative Oncology Group; EFS, event-free survival; HNSCC, head and neck squamous cell carcinoma; HPV, human papillomavirus; HR, hazard ratio; IMRT, intensity-modulated radiation therapy; KM, Kaplan-Meier; LA, locally advanced; OS, overall survival; PFS, progression-free survival; PRISMA, Preferred Reporting Items for Systematic reviews and Meta-Analyses; R/M, recurrent and/or metastatic; RECIST, Response Evaluation Criteria In Solid Tumors; RFS, relapse-free survival; RT, radiotherapy; SLR, systematic literature review; TTE, time-to-event; WHO, World Health Organization.
