# Supplementary material for: Correlation Between Early Time-to-Event Outcomes and Overall Survival in Patients With Locally Advanced Head and Neck Squamous Cell Carcinoma Receiving Definitive Chemoradiation Therapy: Systematic Review and Meta-Analysis
Source: Front Oncol. 2022 Apr 28;12:868490. doi: 10.3389/fonc.2022.868490 (PMC9095900; doi:10.3389/fonc.2022.868490)
Supplement: Supplementary file 1 [file DataSheet_1.docx]

Supplementary Material

# Supplementary Appendix A. Methodology of the systematic literature review

Supplementary Table A1. Search strategy for Embase

| **Line** | **Search term** | **Hits** |
| --- | --- | --- |
| 1 | exp head cancer/ | 1,743 |
| 2 | exp neck cancer/ | 4,038 |
| 3 | exp larynx cancer/ | 21,880 |
| 4 | exp mouth cancer/ | 66,479 |
| 5 | exp pharynx cancer/ | 42,720 |
| 6 | exp hypopharynx cancer/ | 5,384 |
| 7 | exp oropharynx cancer/ | 12,659 |
| 8 | exp larynx tumor/ | 32,294 |
| 9 | exp larynx carcinoma/ | 11,132 |
| 10 | ((mouth or gingival or lip* or palat* or tongue or Laryn* or pharyn* or hypopharyn* or oropharyn* or tonsil* or throat or otorhinolaryngologic or oral or paranasal or maxillary sinus or ethmoid sinus) adj6 (cancer* or carcinoma* or neoplas* or tumor* or tumour* or malignan* or SCC)).mp. | 204,859 |
| 11 | (head adj3 neck adj6 (cancer* or carcinoma* or neoplas* or tumo?r* or malignan* or SCC)).mp. | 109,728 |
| 12 | (H&N adj6 (cancer* or carcinoma* or neoplas* or tumo?r* or malignan* or SCC)).mp. | 2,350 |
| 13 | (HNSCC or HN SCC or SCCHN or SCC HN or HNSC or HNC or OP SCC or OPSCC).mp. | 24,195 |
| 14 | or/1-13 | 310,939 |
| 15 | (advanc* or (stage* adj2 "3") or (stage* adj2 iii) or (stage* adj2 iv) or (stage* adj2 "4") or inoperable or unresectable or nonresectable or N2* or N3* or T3* or T4*).mp. | 1,901,069 |
| 16 | 14 and 15 | 51,128 |
| 17 | Clinical Trial/ | 1,004,250 |
| 18 | Randomized Controlled Trial/ | 657,993 |
| 19 | controlled clinical trial/ | 463,655 |
| 20 | multicenter study/ | 288,140 |
| 21 | Phase 3 clinical trial/ | 53,476 |
| 22 | Phase 4 clinical trial/ | 4,316 |
| 23 | exp RANDOMIZATION/ | 91,061 |
| 24 | Single Blind Procedure/ | 42,661 |
| 25 | Double Blind Procedure/ | 184,193 |
| 26 | Crossover Procedure/ | 67,009 |
| 27 | PLACEBO/ | 366,538 |
| 28 | randomi?ed controlled trial$.tw. | 257,879 |
| 29 | CRT.tw. | 36,030 |
| 30 | (random$ adj2 allocat$).tw. | 46,581 |
| 31 | single blind$.tw. | 26,966 |
| 32 | double blind$.tw. | 220,054 |
| 33 | ((treble or triple) adj blind$).tw. | 1,367 |
| 34 | placebo$.tw. | 326,082 |
| 35 | Prospective Study/ | 685,513 |
| 36 | or/17-35 | 2,521,889 |
| 37 | Case Study/ | 78,294 |
| 38 | case report.tw. | 448,990 |
| 39 | abstract report/ or letter/ | 1,196,929 |
| 40 | Conference proceeding.pt. | - |
| 41 | Conference abstract.pt. | 4,092,515 |
| 42 | Editorial.pt. | 691,463 |
| 43 | Letter.pt. | 1,174,077 |
| 44 | Note.pt. | 850,553 |
| 45 | or/37-44 | 7,288,413 |
| 46 | 36 not 45 | 1,849,565 |
| 47 | 16 and 46 | 7,120 |
| 48 | limit 47 to english language | 6,601 |

Embase 1974 to 2021 May 26; Search executed: May 27, 2021

Supplementary Table A2. Search strategy for MEDLINE

| **Line** | **Search term** | **Hits** |
| --- | --- | --- |
| 1 | exp "Head and Neck Neoplasms"/ | 319,946 |
| 2 | exp Laryngeal Neoplasms/ | 27,970 |
| 3 | exp Mouth Neoplasms/ | 71,011 |
| 4 | exp Pharyngeal Neoplasms/ | 35,167 |
| 5 | exp Hypopharyngeal Neoplasms/ | 3,283 |
| 6 | exp Oropharyngeal Neoplasms/ | 8,857 |
| 7 | ((mouth or gingival or lip* or palat* or tongue or Laryn* or pharyn* or hypopharyn* or oropharyn* or tonsil* or throat or otorhinolaryngologic or oral or paranasal or maxillary sinus or ethmoid sinus) adj6 (cancer* or carcinoma* or neoplas* or tumor* or tumour* or malignan* or SCC)).mp. | 176,427 |
| 8 | (head adj3 neck adj6 (cancer* or carcinoma* or neoplas* or tumo?r* or malignan* or SCC)).mp. | 81,869 |
| 9 | (H&N adj6 (cancer* or carcinoma* or neoplas* or tumo?r* or malignan* or SCC)).mp. | 1,053 |
| 10 | (HNSCC or HN SCC or SCCHN or SCC HN or HNSC or HNC or OP SCC or OPSCC).mp. | 15,504 |
| 11 | or/1-10 | 401,920 |
| 12 | (advanc* or (stage* adj2 "3") or (stage* adj2 iii) or (stage* adj2 iv) or (stage* adj2 "4") or inoperable or unresectable or nonresectable or T3* or T4* or N2* or N3*).mp. | 1,260,936 |
| 13 | 11 and 12 | 44,324 |
| 14 | Randomized Controlled Trials as Topic/ | 144,102 |
| 15 | randomized controlled trial/ | 531,897 |
| 16 | Random Allocation/ | 105,367 |
| 17 | Double Blind Method/ | 164,560 |
| 18 | Single Blind Method/ | 30,241 |
| 19 | clinical trial/ | 528,969 |
| 20 | clinical trial, phase i.pt. | 21,647 |
| 21 | clinical trial, phase ii.pt. | 34,891 |
| 22 | clinical trial, phase iii.pt. | 18,446 |
| 23 | clinical trial, phase iv.pt. | 2,103 |
| 24 | controlled clinical trial.pt. | 94,176 |
| 25 | randomized controlled trial.pt. | 531,897 |
| 26 | multicenter study.pt. | 295,029 |
| 27 | clinical trial.pt. | 528,969 |
| 28 | exp Clinical Trials as topic/ | 357,663 |
| 29 | or/14-28 | 1,430,792 |
| 30 | (clinical adj trial$).tw. | 399,496 |
| 31 | ((singl$ or doubl$ or treb$ or tripl$) adj (blind$3 or mask$3)).tw. | 180,265 |
| 32 | PLACEBOS/ | 35,495 |
| 33 | placebo$.tw. | 225,330 |
| 34 | randomly allocated.tw. | 31,045 |
| 35 | (allocated adj2 random$).tw. | 34,480 |
| 36 | or/30-35 | 679,520 |
| 37 | 29 or 36 | 1,722,288 |
| 38 | case report.tw. | 335,093 |
| 39 | letter/ | 1,136,935 |
| 40 | historical article/ | 363,690 |
| 41 | or/38-40 | 1,818,911 |
| 42 | 37 not 41 | 1,683,062 |
| 43 | 13 and 42 | 7,594 |
| 44 | limit 43 to english language | 7,029 |

Ovid MEDLINE(R) and Epub Ahead of Print, In-Process & Other Non-Indexed Citations and Daily 1946 to May 26, 2021 – Search executed: May 27, 2021

Supplementary Table A3. Search strategy for CENTRAL

| **Line** | **Search term** | **Hits** |
| --- | --- | --- |
| 1 | exp "Head and Neck Neoplasms"/ | 5,929 |
| 2 | exp Laryngeal Neoplasms/ | 320 |
| 3 | exp Mouth Neoplasms/ | 620 |
| 4 | exp Pharyngeal Neoplasms/ | 736 |
| 5 | exp Hypopharyngeal Neoplasms/ | 69 |
| 6 | exp Oropharyngeal Neoplasms/ | 222 |
| 7 | ((mouth or gingival or lip* or palat* or tongue or Laryn* or pharyn* or hypopharyn* or oropharyn* or tonsil* or throat or otorhinolaryngologic or oral or paranasal or maxillary sinus or ethmoid sinus) adj6 (cancer* or carcinoma* or neoplas* or tumor* or tumour* or malignan* or SCC)).mp. | 10,071 |
| 8 | (head adj3 neck adj6 (cancer* or carcinoma* or neoplas* or tumo?r* or malignan* or SCC)).mp. | 8,237 |
| 9 | (H&N adj6 (cancer* or carcinoma* or neoplas* or tumo?r* or malignan* or SCC)).mp. | 233 |
| 10 | (HNSCC or HN SCC or SCCHN or SCC HN or HNSC or HNC or OP SCC or OPSCC).mp. | 2,006 |
| 11 | or/1-10 | 19,162 |
| 12 | (advanc* or (stage* adj2 "3") or (stage* adj2 iii) or (stage* adj2 iv) or (stage* adj2 "4") or inoperable or unresectable or nonresectable or T3* or T4* or N2* or N3*).mp. | 150,888 |
| 13 | 11 and 12 | 6,231 |
| 14 | limit 13 to english language | 3,570 |

EBM Reviews - Cochrane Central Register of Controlled Trials - April 2021 - Search executed: May 27, 2021

Supplementary Table A4. Study eligibility criteria for the systematic literature review

| **Criteria** | **Description** | |
| --- | --- | --- |
| Population | *Inclusion criteria:*   - Patients with newly diagnosed, treatment-naïve, locally advanced HNSCC (regardless of HPV status and tumor location) - Patients not considered for primary surgery based on investigator decision. | |
|  | *Exclusion criteria:*   - Previously treated patients (recurrent cases) - Patients with distant metastasis - Patients with T1/T2 tumors without at least an N2a metastasis (i.e. early-stage, local, non-advanced tumors) - Patients with nasopharyngeal carcinoma | |
| Interventions | *Inclusion criteria* | *Exclusion criteria* |
|  | - RT ± concurrent systemic treatment ± any adjuvant systemic treatment - RT ± induction/neoadjuvant chemotherapy ± any adjuvant systemic treatment - PD1/PD-L1 inhibitors and targeted therapies, as monotherapy or in combination with other treatments specified above | - Surgery ± other treatment modalities - RT alone - Chemotherapy alone - Palliative RT (e.g., re-irradiation for recurrent HNSCC) |
| Comparators | *Inclusion criteria* | *Exclusion criteria* |
|  | - Placebo or best supportive care - Any intervention of interest | - Trials comparing different doses/schedules of the same intervention |
| Time to Event Outcomes | - Overall survival (OS) - Event-free survival (EFS) - Progression-free survival (PFS) - Recurrence-free survival (RFS) - Disease-free survival (DFS) | |
| Time | - No time restriction | |
| Study design | *Inclusion criteria* | *Exclusion criteria* |
|  | - Phase II or phase III randomized controlled trials | - Observational studies - Phase I trials, non-randomized trials, dose-finding/dose escalation/dose-expansion trials - Case series |
| Language | - Publications in English only | |

HNSCC, head and neck squamous cell carcinoma; HPV, human papillomavirus; PD-1/PD-L1, programmed cell death protein 1 or its ligand; RT, radiotherapy.

# Supplementary Appendix B. Technical details of the statistical analysis

The basic model was a linear regression of $\ln\left( {HR}_{OS} \right)$ on $\ln\left( {HR}_{EFS} \right)$:

| ${\ln\left( {HR}_{OS} \right)}_{i}=\beta_{0}+\beta_{1}{ln({HR}_{EFS})}_{i}$, | (1) |
| --- | --- |

where *i* indexes trial. To account for uncertainty in observed effects, observations, i.e. reported trial measures, in this regression were weighted by the sample size of the trial.

The utility of an outcome as a good surrogate measure requires that the intercept parameter ($\beta_{0})$ be sufficiently close to zero and the slope parameter ($\beta_{1})$ be significantly different from zero.(1, 2) The intercept parameter close to zero implies that if there is no treatment effect on the surrogate outcome, then no effect is also observed on the true endpoint, whilst the slope parameter establishes a relationship (positive or negative) between treatment effects on the two outcomes. As such, both the intercept and slope parameters resulting from the model were reported. A weighted Pearson’s correlation coefficient was used as measure of the strength of the relationship between treatment effects on EFS and OS in terms of the ln(HRs).

Sensitivity analyses included an interaction term in the linear regression model to account for interaction between other variables that may affect the relationship of EFS with OS. In particular, we considered binary variables, $C_{i}$, which were, a priori, thought to affect the relationship. Here, if either of the additional terms in the model were found to be significant, the surrogacy relationship within the subsets was deemed to be different and further considerations were necessary:

| ${\ln\left( {HR}_{OS} \right)}_{i}=\beta_{0}+\beta_{1}{ln({HR}_{EFS})}_{i}+ \beta_{2}C_{i}+\beta_{3}\left[ {ln({HR}_{EFS})}_{i} \times C_{i} \right]$ | (2) |
| --- | --- |

Firstly, a statistically significant effect of the binary variable on the intercept (a $\beta_{2}$ that was found to be significant to the model) translated to a non-zero intercept for one or both subsets of trials. Thus, the first condition for the surrogate relationship was brought into question. Secondly, if the addition of the binary variable was found to modify the relationship between EFS and OS, the strength of the relationship was considered separately within each subset. However, if $\beta_{2}$ and $\beta_{3}$ were not found to be significant, the model simplified to the linear regression in **Equation** **1**, i.e. the included variable did not significantly change the relationship between EFS and OS in terms of the ln(HRs) and the conditions for surrogacy were unchanged.

For each trial, HRs of the study treatment relative to the comparator was used to ensure consistency. However, if the study reported HR in the other direction (i.e. HR of the comparator versus study treatment), then the HR was inverted to maintain this consistency.

# Supplementary Appendix C. Summary of trial characteristics of the included studies

Supplementary Table C1. Summary of the trials comparing concurrent chemoradiotherapy to concurrent chemoradiotherapy

| Trial | Interventions | RT modality | RT dose | Masking & phase | Multi-center | Maximum follow-up (months) | Early time-to-event outcome (label and endpoints) | Response assessment criteria |
| --- | --- | --- | --- | --- | --- | --- | --- | --- |
| CONCURRENT CRT VS. CONCURRENT RT + CISPLATIN | | | | | | | | |
| Rischin 2010 (TROG 02.02) (NCT00094081)(3) | RT + tirapazamine + cisplatin | RT | 45-70 | Open-label  Phase III | Yes | 24 | FFS:  Death; persistent disease in the primary site; progression of disease in the neck in patients not undergoing neck dissection; residual disease left behind after neck dissection; locoregional relapse after complete response; distant metastasis | NR |
|  | RT + cisplatin |  |  |  |  |  |  |  |
| Gregoire 2011  (NCT00229723)(4) | RT + cisplatin | 2D- or 3D-RT | 70 | Double-blinded  Phase II | Yes | 24 ^b^ | PFS:  Death; disease progression **^a^** | RECIST |
|  | RT + cisplatin + gefitinib (250) |  |  |  |  |  |  |  |
|  | RT + cisplatin + gefitinib (500) |  |  |  |  |  |  |  |
|  | RT + cisplatin + gefitinib (250) maintenance with gefitinib (250) |  |  |  |  |  |  |  |
|  | RT + cisplatin + gefitinib (500) maintenance with gefitinib (250) |  |  |  |  |  |  |  |
|  | RT + cisplatin maintenance with gefitinib (250) |  |  |  |  |  |  |  |
|  | RT + cisplatin maintenance with gefitinib (500) |  |  |  |  |  |  |  |
| Harrington 2013  (NCT00387127)(5) | RT + cisplatin + lapatinib | IMRT/2D or 3D-conformal RT | 65-70 | Double-blinded  Phase II | Yes | 40 | PFS:  Death; local/disease progression; distant metastases; second primary malignancy | RECIST |
|  | RT + cisplatin + placebo |  |  |  |  |  |  |  |
| Mesia 2015 (CONCERT-1) (NCT00500760)(6) | RT + cisplatin + panitumumab | 3D-RT/IMRT | 70 | Open-label  Phase II | Yes | 38 | PFS:  Death; any recurrence **^a^** | Modified WHO |
|  | RT + cisplatin |  |  |  |  |  |  |  |
| Martins 2013  (NCT00410826)(7) | RT + cisplatin | IMRT/3D-conformal RT | 66-70 | Open-label  Phase II | Yes | 60 | PFS:  Death; disease progression **^a^** | RECIST |
|  | RT + cisplatin + erlotinib |  |  |  |  |  |  |  |
| Ang 2014 (RTOG 0522) (NCT00265941)(8) | RT + cisplatin + cetuximab | IMRT/AFX-CB | 70-72 | Open-label  Phase III | Yes | 60 | PFS:  Death; local, regional, or distant disease progression **^a^** | NR |
|  | RT + cisplatin |  |  |  |  |  |  |  |
| Giralt 2015 (CONCERT-2) (NCT00547157)(9) | RT + cisplatin | IMRT/3D-conformal RT | 70-72 | Open-label  Phase II | Yes | 48 | PFS:  Death; any recurrence; distant metastasis **^a^** | Modified WHO |
|  | RT + panitumumab |  |  |  |  |  |  |  |
| Siu 2016 (HN.6) (NCT00820248)(10) | RT (accelerated) + panitumumab | IMRT | 70 | Open-label  Phase III | Yes | 60 | PFS:  Death; disease progression **^a^** | RECIST |
|  | RT (standard) + cisplatin |  |  |  |  |  |  |  |
| Gillison 2019 (RTOG 1016) (NCT01302834)(11) | RT + cisplatin | IMRT | 70 | Open-label  Phase III | Yes | 60 | PFS:  Death; disease progression **^a^** | NR |
|  | RT + cetuximab |  |  |  |  |  |  |  |
| Sun 2020 (GORTEC 2015­03) (NCT02022098)(12) | RT + cisplatin + debio 1143 | IMRT | 70 | Double-blinded  Phase II | Yes | 24 | PFS:  Death; disease progression (locoregional or distant) **^a^** | RECIST v1.1 |
|  | RT + cisplatin |  |  |  |  |  |  |  |
| Lee 2021 (JAVELIN)  (NCT02952586)(13) | RT + cisplatin + placebo | IMRT | 70 | Double-blinded | Yes | 34 | PFS:  Death; progressive disease **^a^** | RECIST v1.1 |
|  | RT + cisplatin + avelumab |  |  |  |  |  |  |  |
| CONCURRENT CRT VS. OTHER CONCURRENT CRT REGIMENS | | | | | | | | |
| Fietkau 2020 (PacCis-trial) (NCT01126216) | RT + paclitaxel + cisplatin | Reduced RT | 63.6 | Open-label Phase III | Yes | 72 | DFS:  Death; locoregional persistent disease at re-staging; recurrent disease during follow-up; distant metastasis | NR |
|  | RT + 5-FU + cisplatin | Standard RT | 70.6 |  |  |  |  |  |
| Rischin 2005 (TROG 98-02)(14) | RT + tirapazamine + cisplatin | RT | 45-70 | Open-label  Phase III | Yes | 48 | FFS:  Death; persistent disease in the primary site; progression of disease in the neck in patients not undergoing neck dissection; residual disease left behind after neck dissection; locoregional relapse after complete response; distant metastasis. | NR |
|  | RT + PF |  |  |  |  |  |  |  |
| Tao 2018 (GORTEC 2007-01 ) (NCT00609284)(15) | RT + carboplatin + 5-FU + cetuximab | IMRT | 70 | Open-label  Phase III | Yes | 60 | PFS:  Death; first disease progression (locoregional or distant) **^a^** | NR |
|  | RT + cetuximab |  |  |  |  |  |  |  |
| Bourhis 2020 (GORTEC 2015-01) (NCT02707588)(16) | RT + cetuximab | IMRT | 70 | Open-label  Phase II | Yes | NR | PFS:  Death; disease progression (locoregional or distant) **^a^** | RECIST v1.1 |
|  | RT + pembrolizumab |  |  |  |  |  |  |  |

CRT was defined as the combination of RT and any class of systemic therapies, including chemotherapy, targeted therapy or a combination of both.

^a^ Included in the analysis of trials with matching outcome definitions;

^b^ Source: Trial registry information for NCT00229723 available at www.clinicaltrials.gov.

5-FU, fluorouracil; DFS, disease-free survival; FFS, failure-free survival; IMRT, intensity-modulated radiation therapy; NR, not reported; PF, cisplatin + 5-FU; PFS, progression-free survival; RECIST, Response Evaluation Criteria In Solid Tumors; RT, radiotherapy; WHO, World Health Organization.

Supplementary Table C2. Summary of the trials comparing sequential chemoradiotherapy to concurrent chemoradiotherapy

| Trial | Interventions | RT modality | RT dose | Masking & phase | Multi-center | Maximum follow-up (months) | Early time-to-event outcome (label and endpoints) | Response assessment criteria |
| --- | --- | --- | --- | --- | --- | --- | --- | --- |
| SEQUENTIAL CRT VS. CONCURRENT RT + CISPLATIN | | | | | | | | |
| Prades 2010(17) | IC PF followed by RT | RT | 50-70 | Phase III | Yes | 25 | EFS:  Death; locoregional recurrent disease, metastases **^a^** | NR |
|  | RT + cisplatin |  |  |  |  |  |  |  |
| Hitt 2021 (TTCC 2503) (NCT00261703)(18) | RT + cisplatin | Conventional fractionation | 70 | Open-label  Phase III | Yes | 168 | PFS:  Death; disease progression **^a^** | RECIST |
|  | TPF followed by RT + cisplatin |  |  |  |  |  |  |  |
|  | PF followed by RT + cisplatin |  |  |  |  |  |  |  |
| Lim 2020  (NCT01312350)(19) | RT + cisplatin | IMRT/3D-conformal RT | 63-72 | Open-label  Phase II | No | 60 | PFS:  Death; disease progression; secondary cancer | RECIST |
|  | TPF followed by RT + cisplatin |  |  |  |  |  |  |  |
| Haddad 2013 (PARADIGM) (NCT00095875)(20) | RT + cisplatin | RT | 72 | Open-label  Phase III | Yes | 72 | PFS:  Death; disease progression **^a^** | NR |
|  | TPF followed by RT + carboplatin or RT + docetaxel |  | 72 |  |  |  |  |  |
| Takacsi-Nagy 2015  (EUDRACT 2005-001623-11)(21) | IC TPF followed by RT + cisplatin | RT | 50-70 | Phase II | No | 84 | PFS:  Death; disease progression **^a^** | RECIST |
|  | RT + cisplatin |  |  |  |  |  |  |  |
| Pinnaro 1994(22) | IC PF followed by RT | RT | 65-70 | Phase II | No | 78 | PFS:  Death; disease progression **^a^** | NR |
|  | RT + cisplatin |  |  |  |  |  |  |  |
| Merlano 2020 (INTERCEPTOR-GONO) (NCT00999700)(23) | IC TPF followed by RT + cetuximab | IMRT or 3D conformal RT | 70 | Open-label  Phase III | Yes | 100 | PFS:  Death; local progression; occurrence of distant lesions; progression of existing metastases | NR |
|  | RT + cisplatin |  |  |  |  |  |  |  |
| SEQUENTIAL CRT VS. OTHER CONCURRENT CRT REGIMENS | | | | | | | | |
| Ghi 2017  (NCT01086826)(24) | RT + PF or cetuximab | IMRT/3D-conformal RT | 70 | Open-label  Phase II/III | Yes | 60 | PFS:  Death; disease progression; second primary cancer | RECIST |
|  | TPF followed by RT + PF or cetuximab |  |  |  |  |  |  |  |
| Cohen 2014  (NCT00117572)(25) | IC TPF followed by RT + docetaxel + hydroxyurea + 5-FU | IMRT/3D-conformal RT | 150 | Open-label  Phase II | Yes | 72 | RFS:  Death; locoregional or distant failure **^a^** | RECIST |
|  | RT + docetaxel + hydroxyurea + 5-FU |  |  |  |  |  |  |  |
| Paccagnella 2010(26) | RT + PF | Standard fractionated RT | 60-70 | Open-label  Phase II | Yes | 36 | PFS:  Death; disease progression; second primary cancer | RECIST |
|  | TPF followed by RT + PF |  |  |  |  |  |  |  |
| Geoffrois 2018 (GORTEC 2007-02) (NCT01233843)(27) | RT + PF | IMRT/3D-conformal RT | 50-70 | Open-label Phase III | Yes | 72 | PFS:  Death; disease progression (locoregional or distant) **^a^** | NR |
|  | TPF |  |  |  |  |  |  |  |
| Huang 2018(28) | RT + cisplatin + tegafur-uracil + leucovorin | 6-MV X-ray IMRT | 70-76 | Open-label  Phase II | Yes | 72 | PFS:  Death; disease progression; relapse **^a^** | WHO |
|  | IC (cisplatin + tegafur-uracil + leucovorin) followed by RT + cisplatin + tegafur-uracil + leucovorin |  |  |  |  |  |  |  |
| Burtness 2019  (NCT01345669)(29) | RT + cisplatin/carboplatin + afatinib | -- | -- | Double-blinded  Phase III | Yes | 60 | DFS:  Death; disease recurrence; second primary cancer | NR |
|  | RT + cisplatin/carboplatin + placebo |  |  |  |  |  |  |  |

CRT was defined as the combination of RT and any class of systemic therapies, including chemotherapy, targeted therapy or a combination of both.

^a^ Included in the analysis of trials with matching outcome definitions

5-FU, fluorouracil; DFS, disease-free survival; EFS, event-free survival; IC, induction chemotherapy; IMRT, intensity-modulated radiation therapy; NR, not reported; PF, cisplatin + 5-FU; PFS, progression-free survival; RFS, recurrence-free survival; RECIST, not reported; RT, radiotherapy; TPF, docetaxel + cisplatin + 5-FU; WHO, World Health Organization.

Supplementary Table C3. Summary of the trials comparing sequential chemoradiotherapy to sequential chemoradiotherapy

| Trial | Interventions | RT modality | RT dose | Masking & phase | Multi-center | Maximum follow-up (months) | Early time-to-event outcome (label and endpoints) | Response assessment criteria |
| --- | --- | --- | --- | --- | --- | --- | --- | --- |
| Lorch 2011 (TAX 324) (NCT00273546)(30) | TPF followed by RT + carboplatin | RT | 50-74 | Open-label  Phase III | Yes | 120 | PFS:  Death; disease progression **^a^** | Modified WHO |
|  | PF followed by RT + carboplatin |  |  |  |  |  |  |  |
| Vermorken 2007 (EORTC 24971/TAX 323) (NCT00003888)(31) | RT + TPF | RT | 66-74 | Open-label  Phase III | Yes | 54 | PFS:  Death; disease progression; relapse **^a^** | Modified WHO |
|  | RT + PF |  |  |  |  |  |  |  |
| Seiwert 2016(32) | IC (carboplatin + paclitaxel + cetuximab) followed by RT + 5-FU + hydroxyurea + cetuximab | RT | 75 | Open-label  Phase II | Yes | 72 | PFS:  Death; disease progression **^a^** | RECIST |
|  | IC (carboplatin + paclitaxel + cetuximab) followed by RT + cisplatin + cetuximab |  | 72 |  |  |  |  |  |

^a^ Included in the analysis of trials with matching outcome definitions

PF, cisplatin + 5-FU; PFS, progression-free survival; RECIST, Response Evaluation Criteria In Solid Tumors; RT, radiotherapy; TPF, docetaxel + cisplatin + 5-FU; WHO, World Health Organization.

# Supplementary Appendix D. Trials of chemoradiotherapy that were not eligible for the analysis

Supplementary Table D1. Reasons for exclusion of trials that were not included in the analysis

| **Reasons for exclusion** | **Excluded trials** |
| --- | --- |
| HR or KM data were not provided for both OS and EFS (n=8) | Bhattacharya 2014(33) |
|  | Carinci 2001(34) |
|  | De-ESCALaTE(35) |
|  | ESCORT‑N(36) |
|  | Tao 2016(37) |
|  | REACH, cohort 1(38) |
|  | REACH, cohort 2(38) |
|  | Al-Saleh 2019(39) |
| Definition was not provided for EFS (n=9) | Dutta 2013(40) |
|  | ARTSCAN III(41) |
|  | RTOG 97-03(42) |
|  | RTOG 91-11(43) |
|  | Gupta 2009(44) |
|  | Friesland 2019(45) |
|  | Lee 2015(46) |
|  | AGMT-trial(47) |
|  | Gupta 2013(48) |
| EFS definition did not include both death and disease progression as endpoints (n=7) | Essa and Azzam 2010(49) |
|  | HeCOG(50) |
|  | Patil 2019(51) |
|  | Maddalo 2020(52) |
|  | Reddy 2014(53) |
|  | Halim 2012(54) |
|  | Argiris 2016(55) |
| Few events in OS KM data (n=1) | Rodriguez 2015(56) |

EFS, event-free survival; HR, hazard ratio; KM, Kaplan-Meier; OS, overall survival.

# Supplementary Appendix E. Results from the leave-one-out validation analyses

Supplementary Figure E1. Leave-one-out cross-validation for the linear regression between ln(HRs) of EFS and OS in all CRT trials


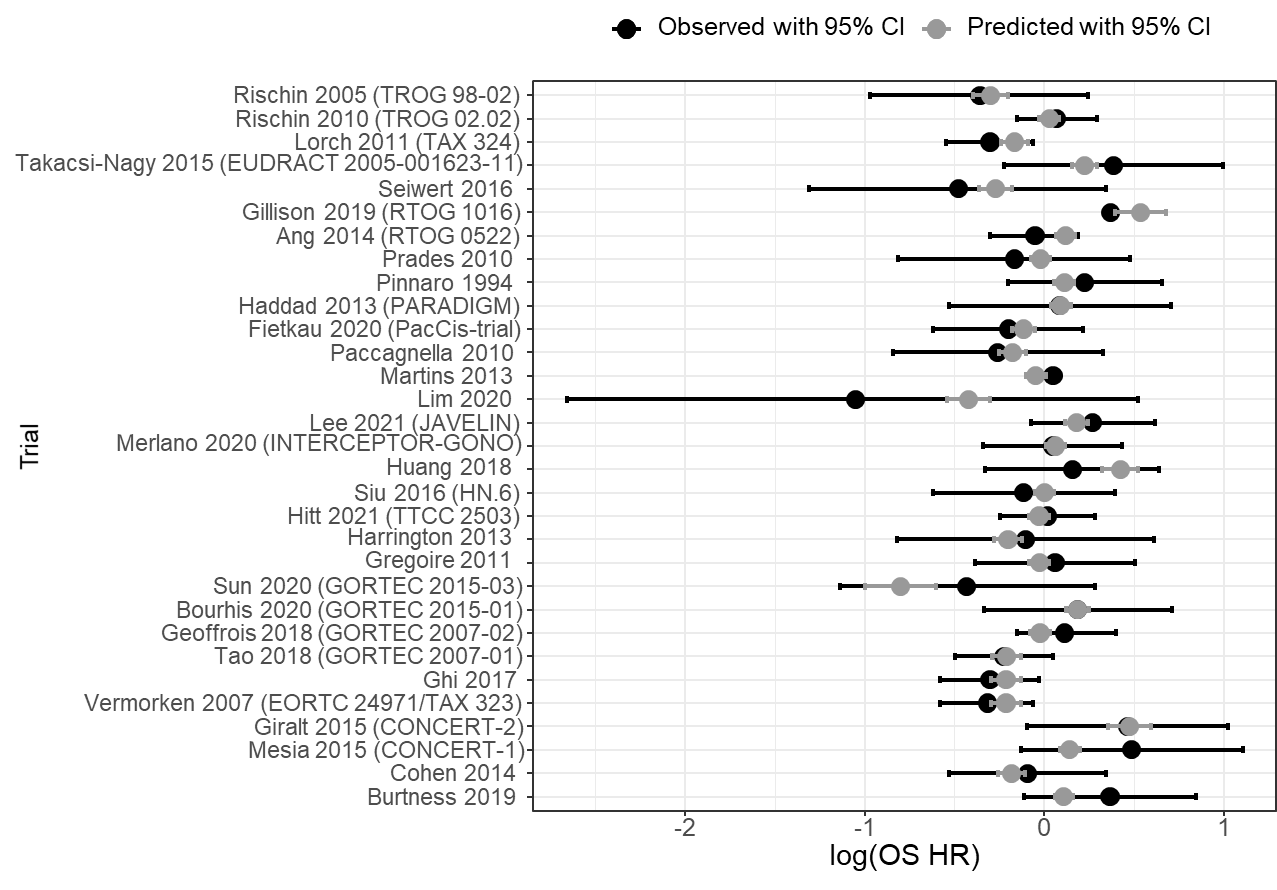


CRT was defined as the combination of RT and any class of systemic therapies, including chemotherapy, targeted therapy or a combination of both.

CI, confidence interval; EFS, event-free survival; HR, hazard ratio; OS, overall survival.

# References

1. Buyse M, Molenberghs G, Burzykowski T, Renard D, Geys H. The validation of surrogate endpoints in meta-analyses of randomized experiments. *Biostatistics* (2000) 1(1):49-67. Epub 2003/08/23. doi: 10.1093/biostatistics/1.1.49. PubMed PMID: 12933525.

2. Papanikos T, Thompson JR, Abrams KR, Städler N, Ciani O, Taylor R, et al. Bayesian hierarchical meta-analytic methods for modeling surrogate relationships that vary across treatment classes using aggregate data. *Statistics in Medicine* (2020) 39(8):1103-24. doi: <https://doi.org/10.1002/sim.8465>.

3. Rischin D, Peters LJ, O'Sullivan B, Giralt J, Fisher R, Yuen K, et al. Tirapazamine, cisplatin, and radiation versus cisplatin and radiation for advanced squamous cell carcinoma of the head and neck (TROG 02.02, HeadSTART): a phase III trial of the Trans-Tasman Radiation Oncology Group. *Journal of Clinical Oncology* (2010) 28(18):2989-95. doi: <https://dx.doi.org/10.1200/JCO.2009.27.4449>. PubMed PMID: 20479425.

4. Gregoire V, Hamoir M, Chen C, Kane M, Kawecki A, Julka PK, et al. Gefitinib plus cisplatin and radiotherapy in previously untreated head and neck squamous cell carcinoma: a phase II, randomized, double-blind, placebo-controlled study. *Radiotherapy & Oncology* (2011) 100(1):62-9. doi: <https://dx.doi.org/10.1016/j.radonc.2011.07.008>. PubMed PMID: 21821303.

5. Harrington K, Berrier A, Robinson M, Remenar E, Housset M, de Mendoza FH, et al. Randomised Phase II study of oral lapatinib combined with chemoradiotherapy in patients with advanced squamous cell carcinoma of the head and neck: rationale for future randomised trials in human papilloma virus-negative disease. *European Journal of Cancer* (2013) 49(7):1609-18. doi: <https://dx.doi.org/10.1016/j.ejca.2012.11.023>. PubMed PMID: 23265705.

6. Mesia R, Henke M, Fortin A, Minn H, Yunes Ancona AC, Cmelak A, et al. Chemoradiotherapy with or without panitumumab in patients with unresected, locally advanced squamous-cell carcinoma of the head and neck (CONCERT-1): a randomised, controlled, open-label phase 2 trial. *The lancet* (2015) Oncology. 16(2):208-20. PubMed PMID: CN-01043406.

7. Martins RG, Parvathaneni U, Bauman JE, Sharma AK, Raez LE, Papagikos MA, et al. Cisplatin and radiotherapy with or without erlotinib in locally advanced squamous cell carcinoma of the head and neck: a randomized phase II trial. *Journal of Clinical Oncology* (2013) 31(11):1415-21. doi: <https://dx.doi.org/10.1200/JCO.2012.46.3299>. PubMed PMID: 23460709.

8. Ang KK, Zhang Q, Rosenthal DI, Nguyen-Tan PF, Sherman EJ, Weber RS, et al. Randomized phase III trial of concurrent accelerated radiation plus cisplatin with or without cetuximab for stage III to IV head and neck carcinoma: RTOG 0522. *Journal of Clinical Oncology* (2014) 32(27):2940-50. PubMed PMID: 25154822.

9. Giralt J, Trigo J, Nuyts S, Ozsahin M, Skladowski K, Hatoum G, et al. Panitumumab plus radiotherapy versus chemoradiotherapy in patients with unresected, locally advanced squamous-cell carcinoma of the head and neck (CONCERT-2): a randomised, controlled, open-label phase 2 trial. *Lancet Oncology* (2015) 16(2):221-32. PubMed PMID: 25596659.

10. Siu LL, Waldron JN, Chen BE, Winquist E, Wright JR, Nabid A, et al. Effect of Standard Radiotherapy With Cisplatin vs Accelerated Radiotherapy With Panitumumab in Locoregionally Advanced Squamous Cell Head and Neck Carcinoma: A Randomized Clinical Trial. *JAMA Oncology* (2016) 3(2):220-6. PubMed PMID: 27930762.

11. Gillison ML, Trotti AM, Harris J, Eisbruch A, Harari PM, Adelstein DJ, et al. Radiotherapy plus cetuximab or cisplatin in human papillomavirus-positive oropharyngeal cancer (NRG Oncology RTOG 1016): a randomised, multicentre, non-inferiority trial. *Lancet* (2019) 393(10166):40-50. PubMed PMID: 30449625.

12. Sun XST, Y.; Le Tourneau, C.; Pointreau, Y.; Sire, C.; Kaminsky, M. C.; Coutte, A.; Alfonsi, M.; Boisselier, P.; Martin, L.; Miroir, J.; Ramee, J. F.; Delord, J. P.; Clatot, F.; Rolland, F.; Villa, J.; Magne, N.; Elicin, O.; Gherga, E.; Nguyen, F.; Lafond, C.; Bera, G.; Calugaru, V.; Geoffrois, L.; Chauffert, B.; Zubel, A.; Zanna, C.; Brienza, S.; Crompton, P.; Rouits, E.; Gollmer, K.; Szyldergemajn, S.; Bourhis, J. Debio 1143 and high-dose cisplatin chemoradiotherapy in high-risk locoregionally advanced squamous cell carcinoma of the head and neck: a double-blind, multicentre, randomised, phase 2 study. *The Lancet Oncology* (2020) 21(9):1173-87. PubMed PMID: 2007636489.

13. Lee NYF, R. L.; Psyrri, A.; Haddad, R. I.; Tahara, M.; Bourhis, J.; Harrington, K.; Chang, P. M. H.; Lin, J. C.; Razaq, M. A.; Teixeira, M. M.; Lovey, J.; Chamois, J.; Rueda, A.; Hu, C.; Dunn, L. A.; Dvorkin, M. V.; De Beukelaer, S.; Pavlov, D.; Thurm, H.; Cohen, E. Avelumab plus standard-of-care chemoradiotherapy versus chemoradiotherapy alone in patients with locally advanced squamous cell carcinoma of the head and neck: a randomised, double-blind, placebo-controlled, multicentre, phase 3 trial. *The Lancet Oncology* (2021) 22(4):450-62. PubMed PMID: 2011527453.

14. Rischin D, Peters L, Fisher R, Macann A, Denham J, Poulsen M, et al. Tirapazamine, Cisplatin, and Radiation versus Fluorouracil, Cisplatin, and Radiation in patients with locally advanced head and neck cancer: a randomized phase II trial of the Trans-Tasman Radiation Oncology Group (TROG 98.02). *Journal of Clinical Oncology* (2005) 23(1):79-87. PubMed PMID: 15625362.

15. Tao Y, Auperin A, Sire C, Martin L, Khoury C, Maingon P, et al. Improved outcome by adding concurrent chemotherapy to cetuximab and radiotherapy for locally advanced head and neck carcinomas: Results of the GORTEC 2007-01 Phase III Randomized Trial. *Journal of Clinical Oncology* (2018) 36(31):3084-90. doi: <http://dx.doi.org/10.1200/JCO.2017.76.2518>. PubMed PMID: 624617583.

16. Bourhis J, Sire C, Tao Y, Martin L, Alfonsi M, Prevost J, et al. LBA38 Pembrolizumab versus cetuximab, concomitant with radiotherapy (RT) in locally advanced head and neck squamous cell carcinoma (LA-HNSCC): Results of the GORTEC 2015-01 “PembroRad” randomized trial. *Annals of Oncology* (2020) 31:S1168.

17. Prades JM, Lallemant B, Garrel R, Reyt E, Righini C, Schmitt T, et al. Randomized phase III trial comparing induction chemotherapy followed by radiotherapy to concomitant chemoradiotherapy for laryngeal preservation in T3M0 pyriform sinus carcinoma. *Acta Otolaryngol (Stockh)* (2010) 130(1):150-5. doi: <https://dx.doi.org/10.3109/00016480902914080>. PubMed PMID: 19449227.

18. Hitt RI, L.; Lopez-Pousa, A.; Berrocal-Jaime, A.; Grau, J. J.; Garcia-Giron, C.; Martinez-Trufero, J.; Guix, M.; Lambea-Sorrosal, J.; del Barco-Morillo, E.; Leon-Vintro, X.; Cunquero-Tomas, A. J.; Baste, N.; Ocana, A.; Cruz-Hernandez, J. J. Long-term outcomes of induction chemotherapy followed by chemoradiotherapy vs chemoradiotherapy alone as treatment of unresectable head and neck cancer: follow-up of the Spanish Head and Neck Cancer Group (TTCC) 2503 Trial. *Clinical and Translational Oncology* (2021) 23(4):764-72. PubMed PMID: 2005863780.

19. Lim SH, Sun JM, Hong J, Oh D, Ahn YC, Chung MK, et al. Induction chemotherapy followed by concurrent chemoradiotherapy versus CCRT for locally advanced hypopharynx and base of tongue cancer. *Korean Journal of Internal Medicine* (2020) 03:03. PubMed PMID: 32241084.

20. Haddad R, O'Neill A, Rabinowits G, Tishler R, Khuri F, Adkins D, et al. Induction chemotherapy followed by concurrent chemoradiotherapy (sequential chemoradiotherapy) versus concurrent chemoradiotherapy alone in locally advanced head and neck cancer (PARADIGM): a randomised phase 3 trial. *Lancet Oncology* (2013) 14(3):257-64. doi: <https://dx.doi.org/10.1016/S1470-2045(13)70011-1>. PubMed PMID: 23414589.

21. Takacsi-Nagy Z, Hitre E, Remenar E, Oberna F, Polgar C, Major T, et al. Docetaxel, cisplatin and 5-fluorouracil induction chemotherapy followed by chemoradiotherapy or chemoradiotherapy alone in stage III-IV unresectable head and neck cancer: Results of a randomized phase II study. *Strahlentherapie und Onkologie* (2015) 191(8):635-41. PubMed PMID: 25782685.

22. Pinnaro P, Cercato MC, Giannarelli D, Carlini P, Del Vecchio MR, Impiombato FA, et al. A randomized phase II study comparing sequential versus simultaneous chemo-radiotherapy in patients with unresectable locally advanced squamous cell cancer of the head and neck. *Annals of Oncology* (1994) 5(6):513-9. PubMed PMID: 7918123.

23. Merlano MCD, N.; Vecchio, S.; Licitra, L.; Curcio, P.; Benasso, M.; Bagicalupo, A.; Numico, G.; Russi, E.; Corvo, R.; Bruzzi, P.; Interceptor trialists. Phase III Randomized Study of Induction Chemotherapy Followed by Definitive Radiotherapy + Cetuximab Versus Chemoradiotherapy in Squamous Cell Carcinoma of Head and Neck: The INTERCEPTOR-GONO Study (NCT00999700). *Oncology* (2020) 98(11):763-70. PubMed PMID: 32629446.

24. Ghi MG, Paccagnella A, Ferrari D, Foa P, Alterio D, Codeca C, et al. Induction TPF followed by concomitant treatment versus concomitant treatment alone in locally advanced head and neck cancer. A phase II-III trial. *Annals of Oncology* (2017) 28(9):2206-12. PubMed PMID: 28911070.

25. Cohen EE, Karrison TG, Kocherginsky M, Mueller J, Egan R, Huang CH, et al. Phase III randomized trial of induction chemotherapy in patients with N2 or N3 locally advanced head and neck cancer. *Journal of Clinical Oncology* (2014) 32(25):2735-43. doi: <https://dx.doi.org/10.1200/JCO.2013.54.6309>. PubMed PMID: 25049329.

26. Paccagnella A, Ghi MG, Loreggian L, Buffoli A, Koussis H, Mione CA, et al. Concomitant chemoradiotherapy versus induction docetaxel, cisplatin and 5 fluorouracil (TPF) followed by concomitant chemoradiotherapy in locally advanced head and neck cancer: a phase II randomized study. *Annals of Oncology* (2010) 21(7):1515-22. PubMed PMID: 20032123.

27. Geoffrois L, Martin L, De Raucourt D, Sun XS, Tao Y, Maingon P, et al. Induction chemotherapy followed by cetuximab radiotherapy is not superior to concurrent chemoradiotherapy for head and neck carcinomas: Results of the GORTEC 2007-02 Phase III Randomized Trial. *Journal of Clinical Oncology* (2018) 36(31):3077-83. doi: <http://dx.doi.org/10.1200/JCO.2017.76.2591>. PubMed PMID: 624617582.

28. Huang PW, Lin CY, Hsieh CH, Hsu CL, Fan KH, Huang SF, et al. A phase II randomized trial comparing neoadjuvant chemotherapy followed by concurrent chemoradiotherapy versus concurrent chemoradiotherapy alone in advanced squamous cell carcinoma of the pharynx or larynx. *Biomedical Journal* (2018) 41(2):129-36. PubMed PMID: 29866601.

29. Burtness B, Haddad R, Dinis J, Trigo J, Yokota T, de Souza Viana L, et al. Afatinib vs Placebo as Adjuvant Therapy After Chemoradiotherapy in Squamous Cell Carcinoma of the Head and Neck: A Randomized Clinical Trial. *JAMA Oncology* (2019) 13:13. PubMed PMID: 31194247.

30. Lorch JH, Goloubeva O, Haddad RI, Cullen K, Sarlis N, Tishler R, et al. Induction chemotherapy with cisplatin and fluorouracil alone or in combination with docetaxel in locally advanced squamous-cell cancer of the head and neck: long-term results of the TAX 324 randomised phase 3 trial. *Lancet Oncology* (2011) 12(2):153-9. doi: <https://dx.doi.org/10.1016/S1470-2045(10)70279-5>. PubMed PMID: 21233014.

31. Vermorken JB, Remenar E, van Herpen C, Gorlia T, Mesia R, Degardin M, et al. Cisplatin, fluorouracil, and docetaxel in unresectable head and neck cancer. *New England Journal of Medicine* (2007) 357(17):1695-704. PubMed PMID: 17960012.

32. Seiwert TY, Melotek JM, Blair EA, Stenson KM, Salama JK, Witt ME, et al. Final Results of a Randomized Phase 2 Trial Investigating the Addition of Cetuximab to Induction Chemotherapy and Accelerated or Hyperfractionated Chemoradiation for Locoregionally Advanced Head and Neck Cancer. *International Journal of Radiation Oncology, Biology, Physics* (2016) 96(1):21-9. PubMed PMID: 27511844.

33. Bhattacharya B, Pal S, Chattopadhyay B, Adhikary SS, Basu J, Ghosh T. A prospective randomised controlled trial of concurrent chemoradiation versus concurrent chemoradiation along with gefitinib in locally advanced squamous cell carcinoma of head and neck. *Clinical Cancer Investigation Journal* (2014) 3(2):146-52. doi: <http://dx.doi.org/10.4103/2278-0513.130160>. PubMed PMID: 372859183.

34. Carinci F, Cassano L, Farina A, Pelucchi S, Calearo C, Modugno V, et al. Unresectable primary tumor of head and neck: does neck dissection combined with chemoradiotherapy improve survival? *J Craniofac Surg* (2001) 12(5):438-43. PubMed PMID: 11572248.

35. Mehanna H, Robinson M, Hartley A, Kong A, Foran B, Fulton-Lieuw T, et al. Radiotherapy plus cisplatin or cetuximab in low-risk human papillomavirus-positive oropharyngeal cancer (De-ESCALaTE HPV): an open-label randomised controlled phase 3 trial. *Lancet* (2019) 393(10166):51-60. Epub 2018/11/20. doi: 10.1016/S0140-6736(18)32752-1. PubMed PMID: 30449623; PubMed Central PMCID: PMCPMC6319250.

36. Kumar A, Chakravarty N, Bhatnagar S, Chowdhary GS. Efficacy and safety of concurrent chemoradiotherapy with or without Nimotuzumab in unresectable locally advanced squamous cell carcinoma of head and neck: Prospective comparative study - ESCORT-N study. *South Asian Journal of Cancer* (2019) 8(2):108-11. doi: <http://dx.doi.org/10.4103/sajc.sajc_38_18>. PubMed PMID: 627460881.

37. Tao Y, Ma C, Yin X, Fang X, Liu L. Therapeutic effects of sequential chemoradiotherapy with pemetrexed and cisplatin on locally advanced laryngeal cancer. *Pakistan journal of medical sciences* (2016) 32(5):1126-30. PubMed PMID: CN-01305108.

38. Tao YA, A.; Sun, X.; Sire, C.; Martin, L.; Coutte, A.; Lafond, C.; Miroir, J.; Liem, X.; Rolland, F.; Even, C.; Nguyen, F.; Saada, E.; Maillard, A.; Colin-Batailhou, N.; Thariat, J.; Guigay, J.; Bourhis, J. Avelumab-cetuximab-radiotherapy versus standards of care in locally advanced squamous-cell carcinoma of the head and neck: The safety phase of a randomised phase III trial GORTEC 2017-01 (REACH). *European Journal of Cancer* (2020) 141:21-9. PubMed PMID: 2008382562.

39. Al-Saleh K, El-Sherify M, Safwat R, Elbasmy A, Shete J, Hussein A, et al. Phase II/III Randomized Controlled Trial of Concomitant Hyperfractionated Radiotherapy plus Cetuximab (Anti-EGFR Antibody) or Chemotherapy in Locally Advanced Head and Neck Cancer. *The gulf journal of oncology* (2019) 1(30):6-12. PubMed PMID: 31242976.

40. Dutta S, Ghorai S, Choudhury KB, Majumder A. Radical treatment of locally advanced head and neck cancer with concurrent chemo radiation-cisplatin versus carboplatin: A randomized comparative phase III trial. *Clinical Cancer Investigation Journal* (2013) 2(2):122-7. doi: <http://dx.doi.org/10.4103/2278-0513.113634>. PubMed PMID: 369248771.

41. Gebre-Medhin MB, E.; Engstrom, P.; Cange, H. H.; Hammarstedt-Nordenvall, L.; Reizenstein, J.; Nyman, J.; Abel, E.; Friesland, S.; Sjodin, H.; Carlsson, H.; Soderkvist, K.; Thomasson, M.; Zackrisson, B.; Nilsson, P. ARTSCAN III: A randomized phase III study comparing chemoradiotherapy with cisplatin versus cetuximab in patients with locoregionally advanced head and neck squamous cell cancer. *Journal of Clinical Oncology* (2021) 39(1):38-47. PubMed PMID: 2010489764.

42. Garden AS, Harris J, Vokes EE, Forastiere AA, Ridge JA, Jones C, et al. Preliminary results of Radiation Therapy Oncology Group 97-03: a randomized phase ii trial of concurrent radiation and chemotherapy for advanced squamous cell carcinomas of the head and neck. *Journal of Clinical Oncology* (2004) 22(14):2856-64. PubMed PMID: 15254053.

43. Forastiere AA, Goepfert H, Maor M, Pajak TF, Weber R, Morrison W, et al. Concurrent chemotherapy and radiotherapy for organ preservation in advanced laryngeal cancer. *New England Journal of Medicine* (2003) 349(22):2091-8. PubMed PMID: 14645636.

44. Gupta D, Shukla P, Bisht SS, Dhawan A, Pant MC, Bhatt ML, et al. A prospective comparision of sequential chemoradiation vs concurrent chemoradiation in locally advanced oropharyngeal carcinomas. *Cancer biology & therapy* (2009) 8(3):213-7. PubMed PMID: CN-00761272.

45. Friesland S, Mercke C, Sjödin H, Dobeln Gv, Carstens H, Margolin G, et al. Randomized phase II study with or without induction chemotherapy combined with accelerated high dose radiotherapy and cetuximab in locally advanced unresectable HPV positive squamous cell carcinoma of the head and neck. *Journal of Clinical Oncology* (2019) 37(15_suppl):6077-. doi: 10.1200/JCO.2019.37.15_suppl.6077.

46. Lee KW, Koh Y, Kim SB, Shin SW, Kang JH, Wu HG, et al. A Randomized, Multicenter, Phase II Study of Cetuximab With Docetaxel and Cisplatin as Induction Chemotherapy in Unresectable, Locally Advanced Head and Neck Cancer. *Oncologist* (2015) 20(10):1119-20. PubMed PMID: 26304911.

47. Keil F, Hartl M, Altorjai G, Pecherstorfer M, Mayrbäurl B, Vries Ad, et al. Induction chemotherapy with docetaxel, cisplatin and cetuximab versus docetaxel, cisplatin and 5-fluorouracil followed by radiotherapy with cetuximab for locally advanced or inoperable squamous cell carcinoma of the head and neck: Promising results of a randomized phase II AGMT-trial. *Journal of Clinical Oncology* (2019) 37(15_suppl):6027-. doi: 10.1200/JCO.2019.37.15_suppl.6027.

48. Gupta S, Khan H, Barik S, Negi MP. Clinical benefits of concurrent capecitabine and cisplatin versus concurrent cisplatin and 5-flurouracil in locally advanced squamous cell head and neck cancer. *Drug discov* (2013) 7(1):36-42. PubMed PMID: 23524942.

49. Essa HH, Azzam M. Concurrent chemoradiation in locally advanced head and neck cancers: a comparative study of weekly Paclitaxel versus Cisplatin-based regimen. *Journal of the Egyptian National Cancer Institute* (2010) 22(3):165-73. PubMed PMID: CN-00849608 UPDATE.

50. Fountzilas G, Ciuleanu E, Dafni U, Plataniotis G, Kalogera-Fountzila A, Samantas E, et al. Concomitant radiochemotherapy vs radiotherapy alone in patients with head and neck cancer: a Hellenic Cooperative Oncology Group Phase III Study. *Med Oncol* (2004) 21(2):95-107. PubMed PMID: 15299181.

51. Patil VM, Noronha V, Joshi A, Agarwal J, Ghosh-Laskar S, Budrukkar A, et al. A randomized phase 3 trial comparing nimotuzumab plus cisplatin chemoradiotherapy versus cisplatin chemoradiotherapy alone in locally advanced head and neck cancer. *Cancer* (2019) 125(18):3184-97. doi: <http://dx.doi.org/10.1002/cncr.32179>.

52. Maddalo M, Borghetti P, Tomasini D, Corvo R, Bonomo P, Petrucci A, et al. Cetuximab and Radiation Therapy Versus Cisplatin and Radiation Therapy for Locally Advanced Head and Neck Cancer: Long-Term Survival and Toxicity Outcomes of a Randomized Phase 2 Trial. *International Journal of Radiation Oncology Biology Physics* (2020). doi: <http://dx.doi.org/10.1016/j.ijrobp.2020.02.637>. PubMed PMID: 2005548219.

53. Reddy BK, Lokesh V, Vidyasagar MS, Shenoy K, Babu KG, Shenoy A, et al. Nimotuzumab provides survival benefit to patients with inoperable advanced squamous cell carcinoma of the head and neck: a randomized, open-label, phase IIb, 5-year study in Indian patients. *Oral Oncol* (2014) 50(5):498-505. doi: <https://dx.doi.org/10.1016/j.oraloncology.2013.11.008>. PubMed PMID: 24613543.

54. Halim AA, Wahba HA, El-Hadaad HA, Abo-Elyazeed A. Concomitant chemoradiotherapy using low-dose weekly gemcitabine versus low-dose weekly paclitaxel in locally advanced head and neck squamous cell carcinoma: a phase III study. *Med Oncol* (2012) 29(1):279-84. doi: <https://dx.doi.org/10.1007/s12032-010-9811-x>. PubMed PMID: 21279703.

55. Argiris A, Bauman JE, Ohr J, Gooding WE, Heron DE, Duvvuri U, et al. Phase II randomized trial of radiation therapy, cetuximab, and pemetrexed with or without bevacizumab in patients with locally advanced head and neck cancer. *Annals of Oncology* (2016) 27(8):1594-600. PubMed PMID: 27177865.

56. Rodriguez CP, Adelstein DJ, Rybicki LA, Savvides P, Saxton JP, Koyfman SA, et al. Randomized phase III study of 2 cisplatin-based chemoradiation regimens in locally advanced head and neck squamous cell carcinoma: impact of changing disease epidemiology on contemporary trial design. *Head & Neck* (2015) 37(11):1583-9. PubMed PMID: 24909549.
